# Medicinal plants used in multiple sclerosis patients, prevalence and associated factors: a descriptive cross-sectional study

**DOI:** 10.1186/s12906-024-04587-y

**Published:** 2024-07-22

**Authors:** Naemeh Nikvarz, Behnaz Sedighi, Mehdi Ansari, Shirin Shahdizade, Reyhane Shojaei, Fariba Sharififar

**Affiliations:** 1 Herbal and Traditional Medicines Research Center, Kerman University of Medical Sciences, Kerman, Iran; 2 Neurology Research Center, Kerman University of Medical Sciences, Kerman, Iran; 3 Pharmaceutical Research Center, Institute of Neuropharmacology, Kerman University of Medical Sciences, Kerman, Iran

**Keywords:** Medicinal plants, Multiple sclerosis

## Abstract

**Introduction:**

Multiple sclerosis (MS) is a chronic and debilitating disease that not only leads to disability and associated condition but also impacts one’s ability to maintain a professional life. People’s acceptance and utilization of medicinal plants (MPs) play an important role in managing their treatment process. As a result, this study aims to investigate the use of medicinal herbs among patients with MS.

**Methods:**

A descriptive cross-sectional study was conducted on 150 MS patients who visited a private clinic and the MS Association in Kerman, Iran in 2021. A questionnaire comprising questions about sociodemographic information, disease variables, and aspects of MPs usage was utilized for data collection. Statistical analysis was performed using SPSS version 20 (SPSS Inc., Chicago, IL). The Chi-square test was employed to identify any association between demographic characteristics and MPs usage. To determine the prevalence of plant use in a specific area and the consensus among informants, the use value (UV) and Informant consensus factor (Fic) were calculated.

**Results:**

The study revealed a high prevalence of MPs usage among MS patients. Chamomile (66.6%) and golegavzaban (62.0%) were the most commonly used plants with the highest UV indices (0.88 and 0.82 respectively), while St. John’s wort and licorice were rarely used (0.67% and 4% respectively). Participants cited pursuing a healthier lifestyle as the primary reason for using MPs (24%). St. John’s wort, lavender, and chamomile were the most satisfying plants (100%, 100%, and 53.0% respectively). Chamomile had the highest Fic too. Most patients were motivated to get MPs from their relatives.

**Conclusions:**

Given the widespread use of MPs among MS patients, neurologists should enhance their knowledge in this area to guide patients away from seeking advice from non-professionals. Providing standardized formulations can help prevent potential interactions between MPs and mainstream drugs, thereby improving patients safety and outcomes.

**Supplementary Information:**

The online version contains supplementary material available at 10.1186/s12906-024-04587-y.

## Introduction

Multiple sclerosis (MS) is an autoimmune and inflammatory disease of the central nervous system (CNS) that causes the destruction of the myelin sheath of axons to varying degrees [1]. This disease is pathologically characterized by the triad of inflammation, demyelination, and scarring of multiple areas of the CNS [2]. Epidemiologically, MS is more prevalent in young people and is the second leading cause of disability in young adults after trauma [3]. Approximately three-quarters of patients with MS are women, and around 2.3 million people worldwide are suffering from MS [4, 5]. The prevalence of this disorder in different geographical areas varies from 3.7 to 30 per hundred thousand people, with a higher number of patients in the northern hemisphere [6]. Ebrahimi et al., reported the prevalence of MS in Iran and Kerman in 2013 as 51.52 and 31.5 per hundred thousand people, respectively, while in Tehran, the capital of Iran, in 2006, the prevalence of MS was 79.3 per hundred thousand people. This number increased to 162.38 per hundred thousand in 2019 [7, 8].

The clinical signs and symptoms of MS are variable and are caused by the involvement of sensory, motor, visual, and brainstem pathways. The location and size of the CNS lesions determine the type and severity of symptoms. Among the most common symptoms of the disease are vision disorders, movement problems, numbness and tingling of the limbs, bowel and bladder dysfunction, sexual dysfunction, fatigue, chronic pain, spasms, tremors, hypersensitivity to heat, weakness, and depression [1, 5]. The disease impacts the physical, emotional health, and social life of people causing important socio-economic consequences for the patient and society [9].

Pharmacotherapy is the mainstay of MS treatment, but there is no cure for this disorder. Several studies have shown that 27–100% of patients with MS use complementary and alternative medicine (CAM) in addition to standard drug regimens to control their symptoms [10–13]. Medicinal plants (MPs) are a modality of CAM [11].

The use of herbal resources has always been an attractive option in the treatment of diseases. The potential benefits of plants, such as antioxidant, anticholinesterase, antifungal, and antitumor properties [14–17] have been scientifically evaluated. Research in this field is considered a strategy for drug discovery. A limited number of animal and clinical studies have evaluated the effects of different MPs in the management of MS. Studies on the animal model of MS, experimental allergic encephalomyelitis (EAE), have shown that herbs or herbal preparations like *Zingiber officinale* [18], *Curcuma longa* [19–21], *Nigella sativa* [22, 23], *Crocus sativus* [24, 25], *Panax ginseng* [26], *Vitis vinifera* [27, 28], and MS 14 [29–31] inhibit or modify some underlying inflammatory processes involved in the pathogenesis of EAE and also reduce its clinical signs. Additionally, clinical studies in patients with MS have demonstrated that herbs such as *Ginkgo biloba* [32, 33], *Panax ginseng* [34], *Boswellia papyrifera* [35], *Camellia sinensis* [36], and *Cannabis sativa* [37–40] improve some signs and symptoms of MS such as fatigue and spasticity.

Despite growing evidence of the safety and efficacy of MPs in the management of MS, many MPs have not been studied or have small studies with low methodological quality in this patient population [41]. Self-treatment with these MPs may adversely affect patients and may have important interactions with standard approved drugs administered in the management of MS [42, 43]. Furthermore, some patients believe that because of their natural origin, MPs are safe and do not even inform their physician that they are using them. Studies have demonstrated that 11–35% of patients with MS use herbal medicine [44–47].

Using traditional medicine has a long history in Iran [48], and many Iranian patients use /MPs/ as remedies for different diseases. However, the number of studies that have investigated the frequency of usage of MPs by MS patients in Iran is limited. These studies reported that most patients with MS (65–97%) use MPs [49, 50]. Identifying the frequency and pattern of MP usage by MS patients can help clinicians make patients aware of adverse effects and interactions of MPs with other drugs. Additionally, the experiences of patients regarding the efficacy of each plant in ameliorating their symptoms can be a good opportunity for further research to find more therapeutic options for the management of MS signs and symptoms. So far, few studies have been conducted in Iran to evaluate the self-reported pattern of using MPs in routine visits to modern medicine clinics. Due to the lack of data and information in this field, and the importance of knowing this data due to possible irreversible effects, this study was designed to determine the frequency of and factors associated with MPs usage by MS patients, reasons for using them, and patient experiences regarding the effectiveness of MPs in guiding correct and appropriate prescribing by physicians.

## Methods and materials

### Study design

This descriptive cross-sectional study was conducted from June to July 2021. The Ethics Committee of Kerman University of Medical Sciences reviewed and approved the study protocol (reference number: IR.KMU.REC.1399.158). Written informed consent was obtained from all participants before enrolling in the study.

### Study population

Participants were recruited from the private clinic of the neurologist member of the research team (BS) and the MS Association in Kerman, Iran.

Patients over 18 years old with at least a 6-month history of MS who could communicate and understand questionnaire’s concepts, either independently or with the help of a companion, were included in the study. Patients who died for various reasons or were not eager to continue participating in the study were excluded.

### Sample size determination

To calculate the sample size needed to estimate the proportion of patients with MS who use MPs, the researcher considered a 10% estimate based on the study’s findings on complementary treatments for MS. Using the provided formula, a sample size of 140 individuals was determined with a confidence level of 95% and a maximum error of 5%. Accounting for a non-response rate of 10%, the minimum sample size required was 150 individuals [51].

### Questionnaire

The questionnaire, prepared by the researchers based on previous studies, consisted of 3 sections and 25 questions that extracted sociodemographic information, disease variables, and aspects of the use of MPs. This checklist was completed through direct questioning by the participants. The questionnaire initially underwent face validity assessment by a panel of 10 specialized experts based on word usage, item placement and scoring. Additionally, it was reviewed for further validation, and their feedback was incorporated. To measure the reliability, Cronbach’s alpha was calculated after administering 10 pretest questionnaires using SPSS. The first part recorded patients’ demographic characteristics, including age, sex, marital status, locality, level of education, income level, and employment status. The second part, extracted information about the disease including the type of MS (relapsing-remitting, primary progressive, secondary progressive, and progressive relapsing), duration of the disease, level of disability evaluated by the Extended Disability Status Scale (EDSS) (scores 0-4.5 refers to patients who can walk without any aid, and scores 5-9.5 characterize patients with the impairment in walking) [52], and current medications used to manage MS. The third part asked patients about the reasons for MPs use, the source that recommended them (internet, friends, family members, neurologist or other clinicians, etc.), side effects, the type of MPs that they use, and satisfaction level with them. It is important to note that we did not use a scale to evaluate satisfaction level. The participants were asked to subjectively evaluate the results of using each MP and report their satisfaction level based on perceived efficacy.

The questionnaire was explained to the participants in a quiet and private room, and then the patients’ answers were recorded. Participants were assured that their answers to the questionnaire would not impact their routine treatment of MS and that the research was for scientific purposes only. Completing the questionnaire took approximately 10–20 min, and it was ensured that the patients completely and correctly understood the questions (a maximum of 20 min was taken for patients who had less ability to read or understand the questionnaire).

### Use value (UV) and informant consensus factor (Fic)

The prevalence of each medicinal plant’s use among all participants using MPs was calculated using this formula: UV = N_ur_/N_i_. Here, N_ur_ represents the number of plant use reports in the studied population, and N_i_ presents the total number of informants that used at least one medicinal plant. To determine homogeneity among informants regarding the plants to be used for reported symptoms, the Fic was calculated using the following formula: Fic = (N_ur_-N_t_)/(N_ur_-1). In this formula, N_ur_ refers to the number of use-reports per symptom, and N_t_ refers to the number of plant species that were used overall.

### Ethical considerations of research

This study received approval from the ethics committee of Kerman University of Medical Sciences with the ethics code IR.KMU.REC.1399.158. Prior to participating in the study, patients were provided with an explanation of the study’s condition. Upon expressing their consent, patients were able to enter the study. It was also made clear to them that they had the option to withdraw from the study at any point for any reason. Furthermore, patients details were kept confidential and not disclosed anywhere.

### Statistical analysis

Statistical analysis was done using SPSS version 20 (SPSS Inc., Chicago, IL). Continuous data are presented as mean (SD), and categorical data are presented as number (percentage). To compare the demographic characteristics of participants to find any association between these factors and MP usage, the Chi-square test was used. The level of significance was considered < 0.05.

## Results

### Demographic and clinical characteristics of the participants

One hundred fifty patients enrolled in the study. The mean age of the participants was 36.3 ± 5.9 years, and 72% of them were female. The demographic characteristics of the participants are presented in Table 1.

**Table 1:** Demographic characteristics of the patients

Demographic factor	MPs users (*n* = 113) *n* (%)	MPs non-users (*n* = 37) *n* (%)	*P*
Age (year)			
Less than 30	15 (13.3)	4 (10.8)	
30-40	102 (90.3)	24 (64.9)	0.544
More than 40	28 (24.8)	9 (24.3)	
**Sex**			
Female	83 (73.4)	25 (67.6)	0.279
Male	30 (26.5)	12 (32.4)	
**Marital status**			
Married	106 (93.8)	33 (89.2)	0.258
Unmarried	7 (6.2)	4 (10.8)	
**Education level**			
Illiterate	4 (3.5)	1(2.7)	
Diploma and lower	55 (48.7)	15 (40.5)	0.738
Bachelor’s degree and higher	54 (47.8)	21 (56.8)	
**Occupation**			
Employed	54 (47.8)	26 (70.3)	0.009
Unemployed	59 (52.2)	11 (29.7)	
**Income status**			
High	56 (49.5)	19 (51.4)	0.900
Middle	52 (46)	15 (40.5)	
Low	5 (4.5)	3 (8.1)	

Regarding the clinical characteristics, 126 (84%) patients had relapsing-remitting MS, and 71 (47%) patients had a 2–5 year history of MS. The majority of patients (*n* = 67, 44.7%) were using interferon beta-1a as the disease-modifying therapy. Gabapentin was the most common medication used for symptom management of MS (Table 2).

**Table 2:** Clinical characteristics of the patients

**Characteristic**	**Number (%)**
Type of MS	
Relapsing-remitting	126 (84)
Primary progressive	18 (12)
Secondary progressive	3 (2)
Progressive relapsing	3 (2)
**EDSS Score**	
0-4.5	112 (74.7)
5-9	15 (10)
Not evaluated	23 (15.3)
**History of MS**	
Less than 2 years	47 (31.3)
2- < 5 years	71 (47.3)
5-10 years	28 (18.7)
More than 10 years	4 (2.7)
**Last MS attack**	
1-2 weeks ago	23 (15.3)
3-4weeks ago	12 (8)
5-6 weeks ago	6 (4)
8-7 weeks ago	12 (8)
2 months to 1 year ago	34 (22.7)
1-4 years ago	53(35.3)
More than 4 years ago	10 (6.7)
**Disease-modifying therapies**	
Interferon beta-1a	67 (44.7)
Fingolimod	27 (18)
Natalizumab	14 (9.3)
Glatiramer acetate	12 (8)
Dimethyl fumarate	11 (7.3)
Teriflunomide	8 (5.3)
Rituximab	8 (5.3)
Interferon beta-1b	3 (2)
**Drugs for symptom management of MS**	
Gabapentin	99 (66)
Baclofen	33 (22)
Sertraline	22 (14.6)
Imipramine	19 (12.7)
Escitalopram	17 (11.3)
Tizanidine	15 (10)
**Comorbidities**	
Major depressive disorder	13 (8.7)
Generalized anxiety disorder	6 (4)
Diabetes mellitus	5 (3.3)
Hypertension	4 (2.7)
Hyperlipidemia	3 (2)

### Frequency of MP use and association with demographic characteristics

The results of the history of taking herbal medicines showed that 113 (75.3%) participants used at least one medicinal plant in the past three months to manage MS symptoms. The remaining patients (24.7%) did not use any medicinal plant after the diagnosis of MS. The majority of patients (*n* =41, 36.3%) started taking at least one MP in the first months after the diagnosis of MS. In contrast, 20 (17.7%), 26 (23%), and 26 (23%) patients used medicinal plants 6–12 months, 13–24 months, and more than 24 months after being diagnosed with MS, respectively.

Considering the association between demographic characteristics and the use of MPs, the only factor that was significantly associated with MPs usage was employment status. The frequency of unemployed patients reporting MP use was significantly higher than employed participants (Table 1).

The most frequently used herbs by MS patients were chamomile (66.67%) and golegavzaban, (62.0%). On the other hand, St. John’s wort and licorice were rarely used in this sample of patients (0.67 and 4% respectively) (Table 3).

### Reasons for using MPs and the level of satisfaction of participants

The participants were asked to report the reasons for using MPs. Most patients were using MPs to lead a healthier life (24%). Other reasons included decreasing the number of relapse episodes (22.7%), recommendations from friends and relatives (19.3%), safety of MPs and lack of adverse effects (4%), increasing the efficacy of disease-modifying treatments (2%), actively engaging in the course of treatment (2%), decreasing adverse effects of disease-modifying treatments (0.7%), and other reasons (0.7%) (Data not shown).

Regarding the level of satisfaction of participants with using MPs, 53% of those using chamomile were completely satisfied. Unsatisfaction with using an MP was not reported for any herbs except for ginseng and valerian which did not result in expected efficacy in 7.41% and 5.0% of participants, respectively. In Iran, *Nardostachys jatamansi* is offered instead of valerian in *“Attaries”* and the general public have been using this plant for many years (Table 3).

**Table 3:** Frequency and type of medicinal plants used by MS patients and the level of satisfaction of using them

Plant	users (%)	Level of satisfaction				
		Completely satisfied	Relatively satisfied,	Relatively unsatisfied,	Completely Unsatisfied,	No use (%)
		n (%)	n (%)	n (%)	n (%)	
Chamomile	100 (66.7)	53(53.00)	47(47.00)	0(0.00)	0(0.00)	50 (33.33)
Golegavzaban	93 (62.00)	35(37.63)	58(62.37)	0(0.00)	0(0.00)	57(38.00)
Saffron	64 (42.67)	4(6.25)	60(93.75)	0(0.00)	0(0.00)	86(57.33)
Thyme	49 (32.67)	12(24.49)	37(75.51)	0(0.00)	0(0.00)	101(67.33)
Ginger	47 (31.33)	6(12.77)	41(87.23)	0(0.00)	0(0.00)	103(98.67)
Frankincense	41 (27.33)	9(21.95)	32(78.05)	0(0.00)	0(0.00)	109(72.67)
Ginseng	27 (18.00)	5(18.52)	20(74.07)	2(7.41)	0(0.00)	123(82.00)
Rosa	22 (14.67)	5(22.73)	17(77.27)	0(0.00)	0(0.00)	128(85.33)
Cannabis	20 (13.33)	2(10.00)	18(90)	0(0.00)	0(0.00)	130(86.67)
Valerian	20 (13.33)	5(25.00)	14(70.00)	1(5.00)	0(0.00)	130(86.67)
Ginkgo	11 (7.33)	1(9.09)	10(90.91)	0(0.00)	0(0.00)	139(92.67)
Lavender	10 (6.67)	10(100.00)	0(0.00)	0(0.00)	0(0.00)	140(93.33)
Aloe Vera	9 (6.00)	1(11.11)	8(88.89)	0(0.00)	0(0.00)	141(94.00)
Licorice	6 (4.00)	1(16.67)	5(83.33)	0(0.00)	0(0.00)	144(96.00)
St.John’s	1(0.67)	1(100)	0(0.0)	0(0.0)	0(0.0)	149(99.33)

Most of the participants were motivated to use MPs by their relatives (28.7%). Personal experience (27.9%), social media (11.9%), a traditional medicine specialist (9.9%), “Attaries” (4.6%), a nurse (4.6%), a pharmacist (4%), and their neurologist (3%) were other sources.

The symptoms of MS that were managed by using MPs were anxiety (mostly chamomile, 52%), warm temperament (the dominant plant was saffron,38.7%), insomnia (the highest rate of use belongs to chamomile,41.4%), memory impairment (mostly frankincense, 27.3%), tremor, sexual dysfunction, immune system impairment, relapse episodes, depression, ataxia, and pain. The form of tea bags or raw medicinal plants were favored by more than 87% of patients and were mostly prepared from “*Attari*” “, a store where crude medicinal plants are sold. Only the patients who consulted a physician were referred to a pharmacist to provide their drugs.

To accurately assess the common consumption of each plant in the province, the use value (UV) was calculated for each medicinal plant used by the patients in this study. The index showed a wide fluctuation between 0.88 and 0.05. According to the calculations done, the highest UV values were attributed to chamomile and golegavzaban (0.88 and 0.82, respectively). Following them, were saffron, thyme, ginger, and frankincense plants, with higher relative UV indices compared to other plants used (data not shown).

**Table 4:** Informant consensus factor (Fic) to identify the most frequently used species in reduction of each symptom

Symptom	Number of use reports	Number of plants used	Fic	Most frequently used Medicinal plant (s)
Anxiety	113	6	0.95	Chamomile
Ataxia	3	2	0.50	Cannabis
As warm humor	113	7	0.94	Saffron
Immune system disorders	6	2	0.8	Ginseng
Depressed mood	3	3	0.00	-----
Insomnia	66	2	0.98	Chamomile
Memory disorders	46	2	0.98	Frankincense
MS relapses	6	2	0.8	Ginseng
Pain	1	1	-----	Ginger
Sexual disorders	14	2	0.92	Ginseng
Tremor	17	2	0.94	Cannabis

Table 4 displays the results of determining the Informant consensus factor (Fic) of plants to examine the level of agreement among informants for plants used to treat various symptoms of the disease. Except for depression, which has a value of zero, the range in other cases varies between 0.98 (seen in insomnia and memory enhancement) and 0.5 (in ataxia).

## Discussion

This study was conducted to investigate the rate of using medicinal plants as a modality of CAM in patients with MS in Kerman, a city in the southeast of Iran. The results of this study showed that 75.3% of the participants had used medicinal plants. Different studies demonstrated that 33–70% of MS patients use CAM [53]. Results of a study that evaluated the frequency of CAM usage amongst MS patients (*n* = 300) in southern Iran showed that 99.3% of patients used at least one CAM modality of which herbal medicines and dietary supplements were the most common (97.3%) [49]. Another study conducted in Shiraz, a city in the southwestern of Iran, demonstrated that 67.9% of MS patients used CAM of whom 64.2% used to consume herbal medicines [50]. In an investigation regarding the use of CAM among MS patients, carried out in the United States, it was reported that 84% of the patients used complementary treatments, among which herbal treatments (36%) were more common after diet (59%) and supplements (46%) [54]. A study showed that 64.7% of MS patients in South Australia use CAM including vitamins (81.8%), essential fatty acids (80.7%), minerals (62.5%), herbs (37.2%), etc. Most of the patients did not inform their doctors about the use of complementary treatments [10, 55, 56]. The socio-demographic profile of the participants in the present study also indicates that women outnumbered men, with 108 female participants compared to 42 male participants. Dayapoglu and Huybregts in separate studies in Eastern Turkey and Belgium reported that there was no significant difference in the prevalence of CAM usage between men and women with MS [12, 57]. In the study by Shushtari et al., it was found that female MS patients significantly used CAM treatments more than male patients [58]. The majority of patients in the present study, 139 out of 150 (93%) were married, while only 11 patients (7%) were single. Unmarried individuals generally used MPs less than married individuals. However, a study conducted by Shushtari et al. did not find a significant relationship between marital status and the use of medicinal plants in MS patients. In terms of education level, our results showed that 72 patients (48%) had an education level higher than a diploma. In Dayapoglu’s study, significant differences in terms of the level of education were considered between CAM users and non-users. According to the study by Shushtari et al. the use of MPs was higher among individuals with higher education levels [12, 58]. Huybregts et al., reported that the prevalence of CAM use in Belgium was not associated with education level.

In terms of employment status, 75 patients (50%) were employed, 71 patients (47%) were unemployed, and 4 patients (3%) were retired. Employment in a job that generates income is an indicator of household welfare assets. Our study found a significant connection between the use of MPs and employment, which is inconsistent with the findings of Dayapoglu and Shushtari reports about CAM and MPs users in Eastern Turkey and Iran [57, 58]. It appears that the current trend is to utilize MPs as a part of general health care. In our study, we found that, the families of most unemployed patients provide great financial support to the patients. However, in Iran, MPs are primarily used for outpatient diseases which typically require less costly care. This could explain why the use of these remedies is not significantly dependent on the employment and economic status of individuals in other studies.

According to the results of the aforementioned studies and the present one, it can be concluded that complementary treatment methods are similarly and highly used in areas with different cultures and facilities due to the satisfaction and accessibility of natural materials. The use of medicinal plants in MS patients is approximately the same as that of the general population. In this research, the most important influencing factors in the approach of MS patients to the use of medicinal plants included recommendations from relatives, personal experience, a desire for traditional medicine, and social media platforms such as WhatsApp and Telegram. Notably, the least influential factor was physicians’ recommendation. Most people have turned to MPs to live healthier lives and reduce relapses. Moreover, it is believed that MPs do not cause life-threatening adverse reactions. The results also showed that there is no significant relationship between the consumption of MPs, education level, and gender. In a study, it was shown that the method of using complementary treatment did not have a significant relationship with any of the demographic characteristics of the patients, except for the level of education. Additionally, this investigation suggests that people in the age group of 20–29 years old and people with a disease duration of less than one year used complementary treatments more than others. This may be because young people and those recently diagnosed with the disease are more concerned about the progression of the disease and its complications [55]. Although the satisfaction percentage of patients with St. John’s wort and lavender was higher than other plants (100%), the frequency of using these two plants was very low (0.67% and 6.67% respectively), so perhaps the satisfactory results of these two plants are not valid. However, chamomile and golegavzaban, which were used by 66.67% and 62.0% of patients, respectively, had a higher satisfaction rate than other plants (53% and 37.63%, respectively) and were mostly preferred by the patients. Today, the use of herbal medicines among people suffering from chronic diseases such as MS is increasing. However, these patients do not have enough knowledge about the side effects of medicinal plants. A study conducted in Berlin suggests that treatment with MPs can have side effects and its advantages and disadvantages should be investigated [59]. One of the factors that cause patients to not pay attention to the side effects of MPs may be related to the lack of requirements for regular consumption and specific doses of MPs. On the other hand, due to the patient’s optimism towards the use of MPs, in case of a side effect, the history of the plant’s use is not taken into account and the use of the plant along with other drugs is unintentionally overlooked. One of the plants investigated in this study is chamomile. Most of the patients used this plant because of its anti-anxiety and sleep-inducing properties. In an experiment that investigated the effect of chamomile extract in postmenopausal women, the results indicated that the plant was beneficial in reducing sleep disorders [60] and resulted in 53% satisfaction among the patients. However, the ambiguity surrounding the scientific name of the plant used is a concern. Chamomile is a common name that patients in Kerman and many other parts of Iran use for different genera and species related to *Matricaria chamomila*, whose their scientific name was not possible to be identified and determined. It should be noted that in Iran, several plants of different genera and species are used under the name of chamomile, or the Persian name of “Baboone”, many of which have not been widely studied, for example, “Baboone Shirazi” with the scientific name *Matricaria aurea* or *Tripleurospermum disciforme* and so on. Therefore, not all the used plants can be considered as German chamomile. Although preliminary studies have shown similarities in chemical composition or biological effects [61, 62].

In the present study, nearly 20% of patients were pleased with using ginseng as an immune system enhancer and for the plant’s ability to reduce stress. It appears that due to a lack of information about this plant, it was only used by a few people [63].

Another plant examined in this study is golegavzaban, scientifically known as *Echium amoenum*. According to ancient Iranian medicine, it has a cold nature and can purify the blood, strengthen the kidneys, and reduce stress levels. A study has shown that using golegavzaban caused calmness and reduced anxiety [64]. In this study, patients used this plant for its sedative effects.

In clinical and pharmacological studies, various therapeutic properties have been attributed to saffron (*Crocus sativus*) and its active ingredients, including antihypertensive, anti-depressant, anti-anxiety, anti-convulsant, muscle relaxant, pain reliever, and anti-inflammatory effects. These properties have generated significant interest in investigating the medicinal properties of saffron. Research results have shown that saffron can prevent learning and memory disorders as well as oxidative stress caused by chronic stress [65]. However, in this investigation, most patients used saffron and ginger due to their warm nature and did not consider other potential benefits. It seems that there is still limited awareness of the advantages of these plants among communities. It is important to note the ethnobotanical knowledge of people who may be aware of certain medicinal uses of plants that are not documented in scientific texts, while sometimes the confirmed effects of some plants remain unknown to them [66].

Memory and learning disorders are common issues in stressful situations and aging, which can increase the risks of some diseases like schizophrenia and Alzheimer’s disease. In today’s industrialized society, memory disorders are becoming more prevalent, prompting people to seek ways to prevent related disorders and enhance memory. Frankincense (*Boswellia thurifera)* is one of the plants frequently mentioned in many cultures and ancient medicine [67]. Ancient texts have referred to Boswellia for treating nausea and diarrhea, wound healing, and memory enhancement. In the present study, about 27.33% of patients used this plant for memory impairment, showing relative awareness of the plant’s benefits. The satisfaction rate with the use of the plant was approximately 22%. Patients primarily seek to improve memory likely may need to use this MP for a long term to see any effects on memory.

Lavender can affect various parts of the body. Apart from its pleasant aroma and cosmetic benefts, lavender has extensive effects on the central and peripheral nervous system, including sedative, anticonvulsant, antiepileptic, anti-anxiety, anti-depressant, nerve protection, and fatigue improvement properties [68, 69]. Although a low percentage of patients used this plant (6.67%), all of them expressed complete satisfaction with its use.

Roses are widely used in Iranian medicine. Based on the findings of this study, using rose in various forms can improve sexual performance in both men and women. Despite the optimal effects on sexual function, the exact mechanism is unclear. Some researchers suggest possible mechanisms such as influencing brain neurotransmitters and sexual cells, increasing sexual power, and inducing hormonal changes. Roses also have beneficial effects on relieving depression symptoms at a molecular level. The flavonoids in roses have anti-anxiety and anti-depressant effects [70, 71]. Despite the beneficial effects that roses can have on improving symptoms of MS patients, most patients were unaware of them. The main goal of participants in this study was to enhance sexual performance, and none of the patients expressed complete dissatisfaction.

In this study, two indices, UV and Fic were determined. High indices were found for some plants used by patients, such as chamomile and golegavzaban, A high UV value index indicates widespread use and perceived medicinal activity of these plants [72, 73]. In a study by Sullano et al., the highest UV index was approximately 0.54 followed by 0.47, whereas in our study, chamomile and golegavzaban had UV indices of 0.88 and 0.82 and saffron, thyme, ginger, and frankincense had UV indices of 0.57.0.43, 0.41 and 0.36 respectively, demonstrating the extent of their use. All plants in this study had a high Fic value except for golegavzaban. A high Fic suggest agreement among informants on a plant’s specific category of use. The lowest consensus on the golegavzaban is likely due to its native status in Iran, making it less familiar to informants. This difference in ethnobotany explains why despite a high UV index, there is not unanimous agreement on its use. In a study by Ong and Kim [74], which included the Ati Negritos group in Guimaras, the highest consensus (Fic = 1.00) was reported for “ear diseases” and “factors affecting health status and services”. In a study byTantengco et al. [75]. in Dinalupihan, Bataan, the highest Fic value was for “eye diseases” and  “postpartum diseases” (ICF value = 0.858).

Various medications used to treat nervous system-related diseases have undeniable harmful effects and adverse reactions. The search for suitable alternatives has led to an increase in the use of natural treatments worldwide. This highlights the importance of gaining knowledge about MPs and herbal preparations. Further detailed studies are needed to explore the benefits and side effects of MPs as complementary treatments alongside specific therapies. A stronger relationship between physicians and patients can play a significant role in this area.

## Problems and limitations

Several patients were unable to participate in the study due to issues like depression, fatigue, and hopelessness. The questionnaire, originally scheduled for distribution in March 2019, was postponed due to the outbreak of COVID-19 and was finally conducted in June and July 2021, leading some patients to decline interviews due to the pandemic. Additionally, using the questionnaire as an information-gathering tool may have introduced errors, particularly in terms of accurately identifying the plants used, despite our efforts to minimize this possibility.

## Conclusions

The use of a wide range of CAM methods, especially in Western countries, is not as traditional as the use of MPs in Iran. The general interest in using medicinal plants in Iranian society is high, and sometimes patients have a special desire to use MPs, that may not be approved by their doctor. Lack of up-to-date knowledge about the correct and appropriate use of MPs or CAM treatments may cause significant side effects. An appropriate intervention and the opinions and experiences of neurologists who are in direct and continuous contact with patients can help them in choosing and prescribing herbal products correctly. Following up withpatients who use medicinal plants during treatment and providing strategies to sensitize and educate them and those around them will undoubtedly bring positive results. The results of this study can assist physicians in making informed decisions about cooperating and assisting MS patients in various types of health care. According to patients’ feedback, with scientific evaluation and reference to current scientific documents, specific plants that patients show interest in have been useful in improving the symptoms of the disease as part of their treatment protocol. It should be noted that the demographic information and occupational profile of the patients and their relationship with herbal therapy provide insight into their interests, values, needs, and views. By understanding how the patient perceives their disease, the doctor can enhance the necessary skills to address the disease.

Informing the patients of an intervention reduces the adverse effects and negative consequences of herbal treatments. Specialists need to be familiar with the standard formulations of medicinal plants so that this class of drugs can be provided to patients more safely. Seeking advice from pharmacists with academic knowledge in this field is recommended to prevent arbitrary referrals to non-specialists. Finally, it is believed that conducting research with a larger sample size and spatial diversity of the research locations can provide more accurate results in this field, considering the limitations mentioned in this study. The plant species preferred in this study for reducing or alleviating certain symptoms of the disease are supported by several publications referenced in the text. Cliniciansn can prescribe these plants for their patients.

### Electronic supplementary material

Below is the link to the electronic supplementary material.

Supplementary Material 1

## Data Availability

The datasets used and/or analysed during the current study are available from the corresponding author on reasonable request.
