# Supplementary material for: Medicinal plants used in multiple sclerosis patients, prevalence and associated factors: a descriptive cross-sectional study
Source: BMC Complement Med Ther. 2024 Jul 22;24:278. doi: 10.1186/s12906-024-04587-y (PMC11265095; doi:10.1186/s12906-024-04587-y)
Supplement: Supplementary file 1 — Supplementary Material 1 [file 12906_2024_4587_MOESM1_ESM.docx]

**Questionnaire on the use of medicinal plants in multiple sclerosis patients**

| **Socio-demographic characterization** |
| --- |
| Age: <20  20-40  40-50  50-60  60-70  Sex: male  female  Marital status: single  married  divorced  widower  Locality: town  village  city  Level of education: illiterate  diploma  university  Income level: high  low  average  Employment status: employed  unemployed  retired |
| **Disease characteristics and medications used** |
| - Disease type: Primary progressive  Relapsing-remitting  Secondary progressive Relapsing Progressive - The medications used for MS taken in the last 6 months? - A- Disease-modifying therapies   Interferon beta-1a  Fingolimod  Natalizumab  Glatiramer acetate  Dimethyl fumarate  Teriflunomide  Rituximab  Interferon beta-1b   - B- Associated medications (for symptomatic treatment): Baclofen  Tizanidine  Gabapentin  imipramine  Escitalopram  sertraline  others - Last date of disease relapse? - Disease history? less than 2 years  2-5 years  more than 5 years  more than 10 years - EDSS score? - Presence of other diseases (comorbidities)? Blood pressure  diabetes  depression  anxiety  high blood lipids   Other diseases? |
| **use of medicinal plants in multiple sclerosis patients** |
| - How long after the diagnosis of the disease by the doctor did you start using herbs? Less than one year  1-5 years after disease  5-10 years after disease  more than 10 years - Who recommended you take these drugs? - Personal experience  friends and acquaintances  Neurologist   Pharmacist  Traditional medicine Practitioner  Other specialized doctors  General practitioners  Attari  Nutritionist  Nurse  Information shared in social media  Internet   - The reason for using these medicinal plants?   Just for a healthy life Active participation in the treatment process  Recommendations from other people Increasing the effectiveness of drugs used for MS  Decreasing the frequencies of attacks  No serious side effects of medicinal plants  Reducing the side effects of medications used for MS  Other reasons   - Which symptoms of the disease do you use herbal medicine for?   Anxiety  Insomnia  Depression  Sexual dysfunction  Pain Memory disorder Ataxia  Immune system disorders  Tremor  MS relapse  others   - Have you informed your doctor about the use of these herbs? - Do you believe that herbs may interact with medications prescribed by your doctor? - Do you believe medicinal plants may also have side effects and cause some symptoms to worsen? - Have you had any personal experience with side effects or worsening of the disease when using medicinal plants? - The type of the plant and the level of satisfaction? - **Chamomile**: to help control what symptoms of the disease? Name it (.....................) - Level of satisfaction with the use of the plant: completely satisfied relatively satisfied relatively unsatisfied completely unsatisfied - **Golegavzaban**: to help control what symptoms of the disease? Name it (.....................) - Level of satisfaction with the use of the plant: completely satisfied relatively satisfied relatively unsatisfied completely unsatisfied - **Thyme**: to help control of what symptoms of the disease? Name it (.....................) - Level of satisfaction with the use of the plant: completely satisfied relatively satisfied relatively unsatisfied completely unsatisfied - **Aloe Vera**: to help control what symptoms of the disease? Name it (.....................) - Level of satisfaction with the use of the plant: completely satisfied relatively satisfied relatively unsatisfied completely unsatisfied - **Valerian**: to help control what symptoms of the disease? Name it (.....................) - Level of satisfaction with the use of the plant: completely satisfied relatively satisfied relatively unsatisfied completely unsatisfied - **Ginseng**: to help control what symptoms of the disease? Name it (.....................) - Level of satisfaction with the use of the plant: completely satisfied relatively satisfied relatively unsatisfied completely unsatisfied - **Saffron:** to help control what symptoms of the disease? Name it (.....................) - Level of satisfaction with the use of the plant: completely satisfied relatively satisfied relatively unsatisfied completely unsatisfied - **Ginkgo:**  to help control what symptoms of the disease? Name it (.....................) - Level of satisfaction with the use of the plant: completely satisfied relatively satisfied relatively unsatisfied completely unsatisfied - **Licorice:** to help control what symptoms of the disease? Name it (.....................) - Level of satisfaction with the use of the plant: completely satisfied relatively satisfied relatively unsatisfied completely unsatisfied - **Cannabis**: to help control what symptoms of the disease? Name it (.....................) - Level of satisfaction with the use of the plant: completely satisfied relatively satisfied relatively unsatisfied completely unsatisfied - **Rosa**: to help control what symptoms of the disease? Name it (.....................) - Level of satisfaction with the use of the plant: completely satisfied relatively satisfied relatively unsatisfied completely unsatisfied - **Lavender**: to help control what symptoms of the disease? Name it (.....................) - Level of satisfaction with the use of the plant: completely satisfied relatively satisfied relatively unsatisfied completely unsatisfied - **Ginger:** to help control what symptoms of the disease? Name it (.....................) - Level of satisfaction with the use of the plant: completely satisfied relatively satisfied relatively unsatisfied completely unsatisfied - **Frankincense**: to help control what symptoms of the disease? Name it (.....................) - Level of satisfaction with the use of the plant: completely satisfied relatively satisfied relatively unsatisfied completely unsatisfied |
|  |
